# Radiomics for radiation oncologists: are we ready to go?

**DOI:** 10.1259/bjro.20190046

**Published:** 2020-03-25

**Authors:** Loïg Vaugier, Ludovic Ferrer, Laurence Mengue, Emmanuel Jouglar

**Affiliations:** 1 Department of Radiation Oncology, Institut de Cancérologie de l'Ouest, Nantes - Saint Herblain, France; 2 Department of Medical Physics, Institut de Cancérologie de l'Ouest, Nantes - Saint Herblain, France

## Abstract

Radiomics have emerged as an exciting field of research over the past few years, with very wide potential applications in personalised and precision medicine of the future. Radiomics-based approaches are still however limited in daily clinical practice in oncology. This review focus on how radiomics could be incorporated into the radiation therapy pipeline, and globally help the radiation oncologist, from the tumour diagnosis to follow-up after treatment. Radiomics could impact on all steps of the treatment pipeline, once the limitations in terms of robustness and reproducibility are overcome. Major ongoing efforts should be made to collect and share data in the most standardised manner possible.

## Introduction

Over the past decade, extending beyond the conventional radiological image by analysing large amounts of imaging data in order to guide medical care, has been at the heart of imaging research. “Radiomics”—as it is usually called—refers to the machine-learning treatment of the numerous image-based features (or “radiomic” features) that can be extracted from multimodal medical images, *e.g.* CT, positron emission tomography (PET), MRI or ultrasound, and whose combination with other patient “omics” (*e.g.* clinical, metabolic, genomic or proteomic data) enables predictive or prognostic modelling.^1,2^ One reason for its growing popularity is its potential ability to characterise the internal structure of a tissue—such as tumour heterogeneity. Thanks to the development of efficient learning strategies able to deal with such large amounts of data, the door to precise personalised medicine has been opened, with a view to enhanced patient diagnostic, prognostic and therapeutic outcomes in the near future.^3,4^

While radiomics have clearly emerged as an exciting field of research in the past few years, radiomics-based approaches are still very limited in daily clinical practice. In this review, we focus on the potential improvements in the management of cancer patients by radiotherapy (RT), based on a set of studies which does not claim to be exhaustive but illustrative of the radiation oncologist expectations. After briefly describing the methodological aspects, we review how radiomics could globally help the clinical oncologist, from the diagnosis of the tumour to follow-up after treatment.

## Radiomic analysis methodology

A number of reviews offering a general presentation and the methodology of radiomics and features extraction have already been published.^1,2,4,5^ Numerous features can be extracted from medical images in order to specify the structural heterogeneity of tissues, *e.g.* in tumours. These can be as simple to understand and easy to calculate as the mean or variance of the grey levels inside a region or volume of interest (VOI); or more difficult, such as those using co-occurrence matrices. Some other features are related to the geometric characteristics of the VOIs. Such shape-based features (*e.g.* volume, surface area, sphericity and compactness), are closely linked to the tumour segmentation procedures. In PET images, a derived volume parameter, the total glycolic volume defined as the volume times the mean standard uptake value (SUV_mean_), is also used regularly.^6,7^

The simplest quantitative analyses use descriptive statistical methods, such as *maximum, minimum*, *mean* and *variance* of values derived from the grey-level distribution at a pixel level inside some defined VOI, which are either manually or automatically drawn by an expert or by an automatic algorithm. Radiologists commonly use these features in their daily work to characterise Hounsfield units (HUs) or standard uptake values (SUV) inside the CT or PET images respectively. From these grey-level histograms, more advanced statistical analysis could be conducted to characterise the asymmetry (*skewness*), the “flatness” (*kurtosis*), the disorder (*entropy*) or the uniformity (*energy*) of the grey-level distribution.^4^ Since these values are histogram-based, they are often called first-order image features or histogram features.

Textural features classically involve second- or higher-order statistical descriptors able to identify the spatial relationship between voxels of the same magnitude inside a VOI. The main common approaches use grey-level co-occurrences matrices (GLCM), consecutive voxel with the same grey-level magnitude (called a *run*) matrices (GLRLM) and grey-level tone difference matrices (GTDM).^3^ For each of these approaches, several features can be derived, such as second-order *entropy* or *energy*, or more complex features such as *Short Run Emphasis or Long Run Emphasis*.^3^ It is worth noticing that these second- or higher-order features could be derived directly from raw images or after specific image processing being applied as, *e.g.* image rescaling in order to deal with isotropic voxel size or low-pass band image filtering in order to reduce image noise. More advanced filters can be used in order to capture the existence of more sophisticated repetitive patterns. These *filters* such as Laplacian of Gaussian, Gabor, wavelet and Laws’ mask transforms, tend to track structural edges or borders at different scales of the images.^3,4,8,9^

Another interesting approach is to estimate the *fractal dimension* inside a VOI. This number describes the self-similarity of the structures at a multiscale level. The most commonly used algorithms are box-counting in the case of binary volumes, or differential box counting in the case of grey-level volumes.^10^

The identification and selection of reliable and suitable radiomic features, allow to elaborate prediction modelling whose objective is to support treatment decision-making.^7,8^ To do so, the selected radiomic features known as the radiomic signature, feed a variety of machine learning algorithms in order to derive the best model for the presented data set and outcome.^2^ The performance evaluation of the model is then achieved by splitting the whole data set into a training and validation data set thanks to methods like bootstrapping or cross-validation. Ideally, the validation of the model should be performed with an independent testing data set in order to insure that the model is generic enough to encompass new presented data.

As radiomic studies are strongly related to the modelling of patient outcomes, the evaluation of these studies should be tested and reported according to criteria such as those listed in guidelines like TRIPOD.^11^

## Radiomics: from data to clinical practice

Although useful correlations were reported between radiomic features and other patient “omic” traits or even therapeutic outcomes, several challenges remain to be addressed for radiomics to be more widely used in routine practice.^1^

Firstly, only a few clinical software packages are currently available (Dosisoft, OncoRadiomics, TexRad), although a larger number will undoubtedly emerge in the future. Interestingly, some research tools are free of charge (IBEX, CGITA, CERR, QMaZda, LifeX, Pyradiomics, RaCaT) allowing more easily the spreading of radiomics among medical community. Both, commercial and non-commercial software should be fully tested before use. Indeed, some authors compared radiomic features from in-house and freely packages and showed significant differences mainly in second-order features.^12^ The lack of concordance has been identified coming from algorithm implementations, image pre-processing and naming conventions. A recent review stressed out the lack of standardisation and suggested to test the reproducibility and robustness of radiomic packages.^13^ An international collaboration has recently begun working on a standardisation for extraction of image biomarkers in radiomics.^14^ The authors made regular reports describing the process for acquired and reconstructed medical imaging. Guidelines for radiomic studies and nomenclature of radiomic features were also provided, as well as a digital data set for benchmark purposes (https://github.com/theibsi/data_sets).

Once confidence has been gained in radiomic features calculation, the question of what are the best radiomic features that correlate with other “omics” traits or patient outcomes, remains. In other word, one wants to identify the radiomic signature.

One approach is to use all the radiomic features available and identify those which correlate best with the medical situation. In most cases, the situation is that few patients are considered in studies as compared to the number of extracted radiomic features (>100). Classical statistical inference does not suit this situation well. Usually, the patient population is divided in two groups: a training set and a test set. The best image biomarkers are selected from the training set whilst they are evaluated on the test set, a widely used approach in machine learning methods. Unfortunately, certain radiomic features reflect the same characteristic inside the image, despite a different mathematical expression or calculation being used. The high correlation between features might cause overfitting, then responsible for inadequate performance on the test set. High number of features relatively to the number of patients, is another risk for overfitting. The number of patients in cohorts and the complexity of the model (*i.e.* the number of features) need thus to be balanced.

Choosing *a-priori* a set of features extracted from already published scientific publications and known as uncorrelated, is an option to circumvent the pitfalls mentioned above. Such approach seems appealing but one has to be certain that the radiomic signature previously identified is completely valid for the data set at hand. Common approaches use univariate or multivariate regression analysis for the feature selection, thanks to statistical tests such as Student t-test or Mann–Whitney *U*-test.^2,15^ Dimensionality reduction algorithms such as principal component analysis (PCA), are designed to convert a set of possibly correlated features into a set of new uncorrelated ones.^16^ The least absolute shrinkage and selection operator (LASSO) Cox regression model is also a suitable alternative for the regression of high-dimensional data.^2^

In terms of selecting the features themselves, some, notably textural features, are sensitive to image quality (mostly driven by noise, spatial sampling and grey levels). Standardisation of acquisition and reconstruction is a key concept in order to ensure that image quality remains constant: (i) for a given set of parameters and (ii) and/or over a set of different acquisition systems.^17^ As an example, reproducibility of radiomic features with respect to different image processing algorithms or acquisition/reconstruction parameters, was evaluated using interclass correlation coefficient (ICC) or concordance correlation coefficient (CCC)^18–20^—both traditionally used for assessing reliability between multiple observers. The point is particularly true in the context of multicentre clinical trials where “omics” analysis are conducted.^21^ A large number of radiomic features and values found from other “omics” disciplines (*e.g.* genomics and proteomics) are obtained compared to the number of patients available in a single institution. Multicentre clinical trials partially circumvent this limitation but face the problem of heterogeneous grouped “omics” data. Here again, an *a-priori* standardisation is helpful for this. As an illustration, one European nuclear medicine association is promoting an initiative to standardise tomographic reconstructions (EARL) in order to harmonise quantitative results obtained from PET systems worldwide.^22^ This type of standardisation is more challenging for MRI systems, as the acquisition sequences from different vendors have more differences. Harmonisation is often impossible in multicentre retrospective studies or even single centre trials conducted using several systems, due to the lack of raw data. In these situations, removing known batch effects with statistical approaches such as *Combat*, which were initially developed for high throughput experiments,^23^ is a valuable solution which has recently been used in MRI.^24^

**Figure 1.**
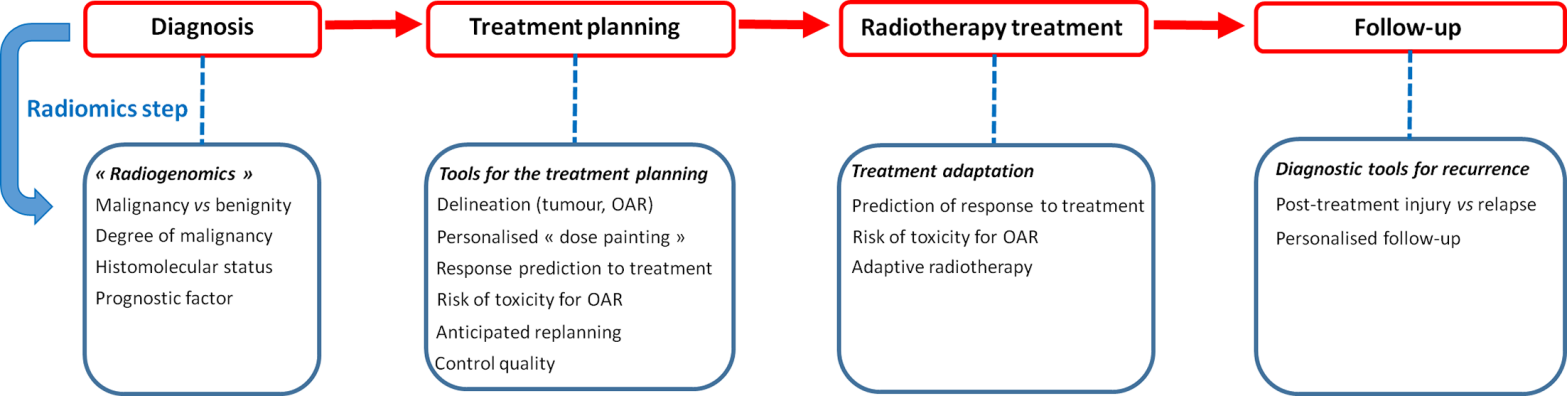
Potential applications of a radiomics step in the radiation therapy pipeline. A non-exhaustive list of examples is shown. OAR, organ at risk.

In the following sections, we focus on a number of studies which illustrate the potential role of radiomics in RT field and the limitations which still exist. The RT pipeline with the benefit input from a radiomic step is summarised in Figure 1. The literature search performed in PubMed until 30 December 2019 and using the keywords “radiotherapy” and “radiomics” or “radiomic” provided 249 entries in total since 2013. We used combinations of the search terms above with “diagnosis”, “radiogenomic”, “dose painting”, “healthy tissue”, “toxicity”. We selected original articles in English which were based on a data set of at least 50 patients and studied radiomic approach in cancer diagnosis, or as a predictive or prognostic factor, or as a tool for RT target volume delineation and prescription, RT control quality, toxicity prediction, adaptive RT and follow-up. The cited references were considered as the most relevant for the clinician oncologists practice. The objective was to illustrate how radiomics may have an impact on the different steps of the RT workflow rather than providing a systematic review of radiomic applications which can be found elsewhere, *e.g.* in Avanzo et al^2^ and Scalco and Rizzo.^5^

## Radiomics for cancer diagnosis

One of the ultimate goal of imaging analysis is to deliver non-invasive diagnoses, although definitive diagnosis has to be provided by histologic analysis. Indeed, by exploring more refined imaging properties in relation with, *e.g*. tumour heterogeneity, tumour radiomics analysis has been shown to be able to capture the biological characteristics of a tumour. Many studies have shown correlations between imaging features and underlying tumour biology at different levels: gene expression or mutations, grade, histological characteristics and microenvironment.

The use of “radiogenomics” to estimate the presence of a specific genomic tumour expression (or damage) known to impact on prognosis or treatment decision, has recently raised particular interest. The term “radiogenomics” is commonly employed in two circumstances. One is referring to the correlation study between patient genetics and response to RT but only the second will be considered below, *i.e*. the relationship between imaging characteristics of tumour and gene expression patterns, gene mutations or other genome-related characteristics.

As examples of this, selection of 110 T2-MRI features allowed an IDH1 status to be estimated with an accuracy of 0.80 in patients with Grade 2 glioma. This figure may even be improved using imaging multimodalities.^25^ A pre-treatment CT radiomic feature (*Law’s Energy*) in a cohort of 47 patients with early-stage non-small cell lung cancer (NSCLC) was significantly predictive for EGFR-mutation status.^8^ Interestingly, this study also showed that the difference in one radiomic feature (*Gabor Energy*) between baseline and an on-treatment CT (3 weeks after beginning the anti-EGFR gefitinib) was associated with mutation status. Tumour volume and maximum diameter were the two other significant predictive parameters, but these only weakly correlated with this radiomic feature. This study demonstrated that a response to targeted therapy did not only produce changes in tumour volume but also changes in the radiomic features. Even though the results need confirmation in a larger population, this study demonstrated that radiomic features may be used as a predictive biomarker.

Pre-treatment radiomic analysis for diagnosis purposes, could have other uses, such as guiding surgery. This was illustrated by Hanania et al who used radiomics to assess the malignant potential of pancreatic intraductal papillary mucinous neoplasms.^26^ While classical imaging criteria incorrectly labelled a lesion as being malignant in 36% of cases, leading to surgical overtreatment, the most predictive radiomic marker (*GLCM* feature) could differentiate low- and high-grade lesions with an area under the receiver operating characteristic curve (AUC) of 0.82, with a sensitivity of 85% and specificity of 68%. Using a cross-validated design on a panel of 10 selected imaging markers, the AUC was even higher (0.96) with a sensitivity of 97% and specificity of 88%.

Radiomics have also been used to evaluate the presence of specific histological substrates. Quantitative radiomic features extracted from breast MRI have been shown to be predictive of breast cancer tumour phenotypes.^27^ In this study, which was performed on 91 MRIs of biopsy-proven invasive breast cancers, imaging signatures were obtained to distinguish between estrogen receptor (ER) positive *versus* ER negative, progesterone receptor (PR) positive *versus* PR negative, human epidermal growth factor receptor 2 (HER2) positive *versus* HER2 negative and triple-negative *versus* other tumours, with AUC values of 0.89, 0.69, 0.65 and 0.67 respectively.

Finally, apart from the tumour phenotype, radiomics have also been tested in order to characterise the tumour environment. In a preliminary study on nine patients, Yin et al showed that imaging features extracted from 18-FDG (fludeoxyglucose) PET coupled to MRI with contrast enhancement correlated closely with tumour microvascular density as evaluated by CD31-immunostaining after nephrectomy in clear cell renal carcinoma.^28^ Combining functional and structural features from PET-MRI showed closer correlations than using PET or MRI alone. Obtaining information about changes in tumour vascularity using a non-invasive method whilst a patient is on treatment could be particularly valuable in the management of renal cancer.

Precision medicine has largely relied on the characteristics provided by the genomic or proteomic analysis on tissue samples, such as biopsies. Biopsies have the disadvantage of being invasive and non-exhaustive in nature. In addition, biopsies reflect only a small percentage of the entire tumour at a given time. The well-reported heterogeneity of tumour phenotypes (both anatomically and over time), is due to branched evolutionary tumour growth.^29^ Nearly 70% of all somatic mutations are absent in all regions of the tumour. Crucially therefore, analysis of a tumour sample could miss or underestimate the genomic landscape of the tumour. Radiomic analysis therefore has several advantages in terms of improving personalised medical decision-making: the wide accessibility of medical imaging, the variety of imaging modalities available (ultrasound, CT, MRI, PET), the ability to take account of the whole tumour and image monitoring during and after treatment. Nevertheless, one of the main reasons for the still limited impact of radiomics on everyday practice may be the difficulty in interpreting the relevant imaging biomarkers (such as the radiomic features) in biological terms.^30^ Future studies will need to investigate how tumour biology and phenotypes translate into imaging features. Furthermore, most of the results need to be validated on large prospective independent cohorts.

## Radiomics as a prognostic and predictive indicator

One major future prospect of radiomics is its development as an important prognostic tool for cancer risk assessment, mostly via quantification of tumour heterogeneity. The predictive power of radiomics has also been investigated in a very large number of studies involving various histological types, *e.g.* glioblastoma, head and neck, lung, cervical, prostate and rectal cancers.^1^ Most of these studies have relied on a pre-treatment multimodal (CT, MRI or PET) analysis. Radiomic parameters were usually combined with clinical factors in order to build accurate predictive models. Various hypotheses have been explored, including risk of local recurrence, distant metastases and overall survival.^9,31,32^ In the future, these radiomic analyses could be performed at baseline as part of a personalised treatment, to help the clinician select the treatment which offers the greatest probability of disease control.

As examples of this, in 2014, Aerts et al. published a radiomics analysis of tumour phenotypes from a data set of 1019 lung and head and neck cancers (HNCs).^33^ Their analysis was based on an extraction of 440 image features and they showed that a large number of these features were of prognostic value. A radiomics signature was designed by selecting the most stable and reproducible features, which were determined by test–retest and interobserver tests. This signature had powerful prognostic value and was validated in three independent cohorts.

The peritumour tissue may also provide information which can be examined by radiomics with benefit in the prediction modelling.^34^ In an analysis of 65 patients with glioblastoma, peritumour features extracted from the oedematous region (T2 and fluid attenuated inversion recovery MRI), were found to have a stronger prognostic value than the features extracted from other regions (*e.g.* tumour contrast-enhanced or necrosis), and could distinguish long- *versus* short-term survival, especially when combined with clinical characteristics (age and Karnofsky performance status).^35^ However, independency between radiomic features and clinical factors was not assessed and no external validation was performed. The potential value of peritumour tissue analysis has also been highlighted in the pre-operative MRI from 63 patients with breast cancer.^36^ The relative volumetric proportion of fat-to-1 cm around the tumour, correlated significantly with positive axillary lymph nodes.

Pre ± per-treatment PET/CT parameters (*e.g.* SUV_max_) have been extensively investigated in order to build predictive models.^15^ In cervical cancer for example, a small subset of PET/CT radiomic features has been found to be of greater value than the SUV_max_ or standard clinical variables to predict local recurrence.^32,37^ The case of neoadjuvant treatments (*e.g.* radiochemotherapy) represents an interesting challenge for prediction modelling, as the definitive answer is provided by histological analysis. The ability to predict complete disease response (as is done, *e.g.* for rectal or oesophageal cancers^38,39^) would have major consequences on further treatments.

This type of radiomic analysis still however needs to be demonstrated in terms of accuracy and reproducibility on larger patient numbers with TRIPOD-based criteria^11^ in order to form a definitive part of the treatment strategy. Testing the relation between radiomic features and other prognostic factors will allow identifying confounding biases. As example, the radiomic signature from Aerts et al^33^ showed high correlation to tumour volume which also strongly impacted prognosis. For this issue, Welch et al^40^ recommended radiomic safeguards as following: the use of an open-source software, detailed methodology, comparison between radiomics and well-known clinical factors, features multicollinearity investigation, test for underlying dependencies between features, data pre-processing and clear segmentation process.

## Radiomics for delineation and dose prescription

Delineation is a delicate and crucial step in RT treatment. Contouring is a manual and time-consuming process that suffers from inter- and intrapractitioner variability. Extracting useful information from modern multimodal imaging could help and/or improve the quality of delineation. Nevertheless, the amount of potential information generated by modern imaging (CT, MRI, PET) is tremendous and challenging to interpret. Automated extraction of imaging features through radiomics processes could help to reduce this variability, at the same time improving the accuracy of contouring.

Radiomics may also help to characterise the risk of lymph node extension. Identification of lymph node metastases and tumour extension outside of lymph nodes is a key factor in the strategic decision, delineation and prescribed dose, particularly in HNC. The performance of determination of extension outside of lymph nodes by CT and human analysis has revealed an AUC of around 0.6.^41^ Kann et al trained a convolutional neural network to predict the presence of lymph node metastases and extension outside of lymph nodes using pre-operative CT features. In the testing phase on an independent cohort, this model predicted lymph node metastases and extension outside of lymph nodes with an AUC of 0.91 in both cases, and negative predictive values of 0.82 and 0.95 respectively. The computed model performed better than a clinical-risk factor regression model and historical assessment based on radiologic criteria.^42^

Radiomics analysis has also allowed identification of specific regions inside the tumour volume. For example, Shiradkar et al used multiparametric MRI from 11 patients with prostate cancer to train a radiomic classifier which was then used to detect the tumour region in 12 patients from another institution.^43^ The results correlated closely with delineation of the cancer determined by an expert radiation oncologist and was consistent with the histological results. A treatment plan for brachytherapy or external beam RT was then generated and was refined by the probability of tumour spatial distribution based on radiomics. This type of radiomics-guided treatment planning resulted in a marginal increase in dose to the surrounding tissue compared to regular treatment.

Radiotherapy using tumour dose painting (DP)^44^ is other attractive option, in which radiomics could make a significant contribution. Rather than prescription of a standard dose, the DP procedure involves adapting the prescribed dose according to the target volume depending on presumed spatial radiosensitivity. A biological target volume for which the dose would be escalated can be defined by incorporating images such as CT, MRI or PET with specific radiomarkers.^45,46^ The dose prescribed is then either standard via subregions which have been manually or semi-automatically segmented (“DP by contours”), or voxel-dependent (“DP by numbers”). In this latter case, the dose at a given voxel depends on the signal intensity of the corresponding voxel. By its ability to capture tumour heterogeneity through multimodal images, radiomics could therefore be extremely helpful.^15^ Most DP clinical trials have been conducted using functional imaging with PET to delineate the biological target volume,^47–50^ based on SUV thresholds or manual delineations. Although the number of patients included is still limited and the clinical benefit of PET-based dose painting still needs to be confirmed, the concept is promising. A radiomics-based approach should form part of future DP trials.

## Radiomics for control quality of dose delivery

Control quality (CQ) of dose delivery is crucial for RT, especially with the emergence of intensity modulated radiation treatment (IMRT) and stereotactic body radiotherapy (SBRT). IMRT and SBRT are very efficient treatments with, *e.g*. high doses delivered to the tumour or its extension while sparing the organs deemed to be at risk (OAR) around, but at a price of precise and reproducible positioning and dose delivery. Patient-specific control quality tests are thus obligatory before starting RT. Phantoms with electronic portal imaging device dosimetry are commonly used for testing the dose delivery of patient-based RT plan.^51^

Radiomics in CQ of IMRT plans, has recently emerged as a potential more robust and reliable procedure than threshold-based passing criteria.^52,53^ As an example, Nyflot et al^53^ have used 23 IMRT plans, each one separated in three subsets: error-free, introduced random multileaf collimator (MLC) error and systematic MLC error, in order to test the ability of error detection. Higher classification accuracy was obtained after training the model by radiomic approaches compared to threshold-based passing criteria. Interestingly, deep learning with convolutional neural networks lead to the best results.

## Radiomics and healthy tissue toxicity and adaptive radiotherapy

The ability to predict treatment toxicity as compared to its benefit is also crucial in the perspective of personalised medicine, particularly with the development of SBRT and re-irradiation treatments. Dosimetric limitation based on normal tissue complication probabilities (NTCP)^54^ for the OAR around the tumour target, has been shown to be successful: as an example of this, the rate of radiation-induced xerostomia in HNC radiotherapy was reduced with IMRT and parotid gland dosimetric limitation.^55,56^ Unfortunately, these limitations fail to prevent all radiation-induced events, as many individual factors need to be taken into account for a given patient (*e.g.* clinical factors, such as age or body mass index and concomitant drugs such as chemotherapy etc.).^57–59^ Anatomical factors also could interact considerably, as significant geometric changes are usually seen during RT, depending on the site: parotid gland shrinkage for example in HNC RT, with a higher initial volume and higher early volume changes as potential imaging biomarkers.^60–62^ This shrinkage should be considered in terms of modelling predicted toxicity, as it could have complex structural and functional consequences leading to, *e.g.* xerostomia.^63–67^ More generally, highly predictive OAR or tumour volume changes during RT would be particularly helpful in the RT workflow, as it would trigger, *e.g.* replanning, which could be better organised or anticipated, or an adjustment to be made to the prescribed dose, with, *e.g.* escalation or de-escalation.^68,69^ These issues lie at the heart of the general adaptive RT framework.^70,71^ Radiomic approaches could therefore represent significant components of this process.^72^

Improvements in machine learning algorithms^73,74^—through the huge amount of data they can process—have made it possible to investigate the hypothesis that OAR radio-induced toxicity is associated with the OAR structural organisation as captured by image analysis. The use of radiomics to identify specific image signatures in healthy tissue and the correlation with radiation-induced toxicity, is a promising emerging area of research,^5,75,76^ which is mostly studied in terms of the risk of xerostomia or radiation pneumonitis centred on the parotid glands^61,66,77,78^ and lung analysis,^79–82^ respectively. Recently, “dosiomic” analysis has also emerged, as an extension of texture analysis but based on the dose distribution which is planned.^66,83,84^ Dosiomic parameters could be interestingly combined to other variables, *e.g.* clinical factors or radiomic parameters, for the construction of more accurate toxicity prediction models. It was applied for the prediction of late urinary and digestive toxicity in prostate RT,^83^ radiation pneumonitis in thoracic RT^84^ and xerostomia in HNC RT.^66^

As an example of all this, Scalco et al^77^ were able to show significant textural changes with RT (*e.g.* the fractal dimension) for the parotid glands in cavum cancer. These changes were suggestive of radiation-induced structural transformation of the glandular tissue, corresponding to less complex organisation after RT. Furthermore, modelling of parotid gland shrinkage after RT has been improved by combining textural changes with the initial volume. Van Dijk et al^78,85,86^ then correlated pre-therapeutic CT and MRI parotid radiomics with the 12 month incidence of xerostomia. They firstly classified heterogeneous parotid glands depending on the baseline fat-to-functional parenchymal tissue ratio using textural parameters. Secondly, they demonstrated that incorporation of the defined heterogeneity improved the model’s performance to predict xerostomia. Parotid textural features have been combined beneficially with demographic factors and dosiomic dose-shape features in order to build more sophisticated NTCP models for xerostomia.^66^ Parotid- and dose-shape features have been shown to be more predictive of long-term xerostomia than the mean parotid dose, especially for the low-dose regimen typically obtained with IMRT.

Similarly for lung tissue, Avanzo et al^87^ have shown that the correlation between voxel-to-voxel density before and after thoracic IMRT, increased with dose. Cunliffe et al^79^ have investigated the ability of radiomics to characterise healthy lung tissue using chest CT and to predict the development of radiation pneumonitis (RP)—a mild to severe lung radiation response. Based on two diagnostic CT scans (one before and one after RT) and the planned dose mapping obtained from 115 patients treated for oesophageal cancer, they found a relationship between dose and the pre- and post-RT change in 20 extracted features in the first-order, fractal, Law’s filter and GLCM classes. Interestingly, 12 features changed significantly in patients who developed RP. Such a texture-based classifier could be used to predict the lung response to radiation, and even the intensity (mild to severe) of this response.^80^ A recent study by Krafft et al^82^ on 192 patients treated for NSCLC) confirmed the gain in RP prediction using radiomic features extracted from pre-RT chest CT scans. CT-based lung texture parameters could also be combined with lung 18 FDG-PET SUV in order to improve the classification performance of RP in a multimodal modelling approach.^88^

A more limited number of studies has also shown potentially useful preliminary results for CT-based prediction of radiation-induced changes in breast tissue during accelerated partial breast irradiation,^89^ sensorineural hearing loss in HNC radiochemotherapy^90^ ; and for MRI-based prediction of trismus in HNC RT.^91^

The potential advantages of general radiomic-based textural analysis to predict toxicity are therefore wideranging and need further investigation in order to be extended into the physicians’ practice. This would help to select patients who will benefit most from highly conformational and/or adaptive RT, *e.g*. in cases carrying a high risk of toxicity.

## Radiomics to discriminate between radiation damage and tumour relapse

Better characterisation of radiation damage is also crucial in the follow-up phase after RT, as discrimination between benign damage and tumour recurrence can be extremely difficult in a standard radiological analysis such as using the RECIST criteria. Radiomic analysis has shown very promising results in this field, especially in lung^92^ and brain disease.^93–95^

As an example of this, in lung SBRT for NSCLC, Mattonen et al^81,92^ have demonstrated the ability of quantitative CT analysis to provide early prediction of recurrence *versus* radiation damage. This analysis was based on higher-density HU consolidation changes and GCLM-derived texture modifications detected as early as 2–5 months after SBRT. When physician- *versus* radiomics-based analysis were compared the median sensitivity and specificity for physician assessment of recurrence were high (around 83 and 75% respectively) although a long average time was required for the first correct detection of a local recurrence (around 15.5 months), and considerable interobserver differences were found. In contrast, the radiomic signature consisted of five features extracted from the ‘periconsolidative and consolidative region’ using semi-automated segmentation. This approach correctly classified 76% of patients, using only with a post-SBRT CT scan within 6 months. A Phase II trial (MISSILE, NCT 02136355) combining SBRT with surgery in NSCLC will provide additional data to test the validity of these radiomics-based approaches, as the definitive distinction between damage and recurrence is provided by the histological examination.

In the case of glioblastoma, a recent classifier^96^ using radiomic features extracted from perfusion MRI images achieved sensitivity of around 90% and specificity of around 88% in differentiating between pseudoprogression and progressive disease.

This type of non-invasive approach to discriminate between benign damage and progressive disease would therefore be extremely welcome for follow-up after RT.

## Conclusions

Sophisticated image analysis such as radiomics could have a tremendous impact on the daily practice of a clinician oncologist, from the diagnosis, type and control quality of treatment, predictions of outcomes and toxicity, through to follow-up after treatment. Radiomic approach however is still not used in the treatment pipeline of radiotherapy. Many barriers have to be overcome before its use becomes widespread in routine practice, *e.g*. regarding the homogenisation of modelling or the meaning and connection of image-based features with biology. Current efforts towards: (i) more homogeneous imaging procedures in order to achieve more reproducible modelling, and (ii) the sharing of data between centres in order to form very large cohorts, and between scientists and physicians in order to test and validate radiomics-based hypotheses, will be crucial for the development of radiomics in the future. Restraints by the clinician community are probably due to the “black box” feeling arising from the machine learning-based approaches, which tend to be even more enhanced, *e.g*. with deep learning. Well-conducted trials would help the clinician appropriating the technique. General machine-learning-based algorithms should not be considered as substitutes to the clinician but rather as efficient tools for personalised medicine performed by “ready-to-go” clinicians.
